# Genetic associations with alcohol drinking initiation and cessation in sub-Saharan African and African ancestry populations

**DOI:** 10.21203/rs.3.rs-10974327/v1

**Published:** 2026-09-09

**Authors:** Jean-Tristan Brandenburg, Ritah Nabunje, Opeyemi Soremekun, Wenlong Carl Chen, Dylan Kerrigan, Christopher G. Mathew, Martin D. Tobin, Tinashe Chikowore, Segun Fatumo, Michèle Ramsay, Chiara Batini, Palwendé Romuald Boua

**Affiliations:** Sydney Brenner Institute for Molecular Bioscience, Faculty of Health Sciences, University of the Witwatersrand; Genomic Epidemiology and Public Health / Genomic Diversity Research Group, MRC/UVRI & LSHTM Uganda Research Unit; Genomic Epidemiology and Public Health / Genomic Diversity Research Group, MRC/UVRI & LSHTM Uganda Research Unit; Sydney Brenner Institute for Molecular Bioscience, Faculty of Health Sciences, University of the Witwatersrand; School of Criminology, Sociology and Social Policy, University of Leicester; Sydney Brenner Institute for Molecular Bioscience, Faculty of Health Sciences, University of the Witwatersrand; Division of Public Health and Epidemiology, School of Medical Sciences, University of Leicester; Sydney Brenner Institute for Molecular Bioscience, Faculty of Health Sciences, University of the Witwatersrand; Genomic Epidemiology and Public Health / Genomic Diversity Research Group, MRC/UVRI & LSHTM Uganda Research Unit; Sydney Brenner Institute for Molecular Bioscience, Faculty of Health Sciences, University of the Witwatersrand; Division of Public Health and Epidemiology, School of Medical Sciences, University of Leicester; Clinical Research Unit Nanoro, Institut de Recherche en Sciences de la Santé

**Keywords:** Alcohol use, Alcohol drinking initiation, Alcohol drinking cessation, Genome-wide association study (GWAS), African ancestry, Sub-Saharan Africa

## Abstract

Alcohol misuse is a major global health concern with significant genetic contributions, yet studies in African populations remain limited. We conducted genome-wide association meta-analyses for alcohol drinking initiation and cessation across multiple cohorts comprising individuals from sub-Saharan Africa (N = 20,936) and those with African ancestry from UK Biobank (N = 7,726). Our analyses identified two genome-wide significant loci associated with drinking cessation (*FYB2* and *FTO* regions) and several suggestive associations overlapping with genes implicated in alcohol metabolism, behavioral traits, and neurodevelopment. These findings provide insights into the genetic architecture of alcohol use behaviors in diverse African populations. By focusing on alcohol drinking initiation and cessation phenotypes, this study complements prior research on alcohol dependence and consumption, highlighting genetic factors influencing vulnerability and resilience. Our results underscore the importance of including diverse ancestries in genetic studies to enhance understanding of alcohol-related behaviors and inform future investigations into prevention and intervention strategies.

## Background

Alcohol use is an integral part of many cultural and social practices, and worldwide ~ 2.3 billion adults are current drinkers^1^, with significant variation in consumption levels across countries and regions^2,3^. However, alcohol consumption is the third leading risk factor for disease and disability in the world, contributing to cancer, cardiovascular diseases, digestive disorders, accidents and violence, and HIV infection^4^.

In Sub-Saharan Africa, the prevalence of alcohol use differs widely by region, age group, and context. Prevalence of lifetime exposure among adults has been reported to range from 34.9% to 86.4%^5^. Factors influencing alcohol use are multifaceted and include social, cultural, demographic, and economic determinants. For example, urbanization, familial context, social stress, and cultural norms are particularly relevant in shaping consumption patterns across countries and regions^5–8^.

Alcohol behaviors and alcohol consumption patterns in sub-Saharan Africa are shaped by complex cultural, socio-economic, and institutional factors. Therefore, we examined alcohol use and alcoholism through a cultural lens, by analyzing literature from the year 2000 onward. Focusing on socio-economic, institutional, and individual cultural influences on drinking behaviors, we noticed significant variation across countries, with religious beliefs, poverty, urbanization, gender norms, and traditional practices influencing consumption and alcohol-related harms. Studies have reported particularly high levels of alcohol use disorders in South Africa, while high levels of abstention in some Burkina Faso populations have been associated with religious sanctions. We highlighted alcohol use as a culturally embedded behavior shaped by broader social and economic contexts^9–13^.

Genetic factors also contribute substantially to alcohol use and dependence. Twin studies, such as FinnTwin and the Virginia Adult Twin Study of Psychiatric and Substance Use Disorders, have demonstrated a strong heritable component in alcohol-related behaviors, pointing to a complex genetic architecture^14,15^. These studies highlight polygenic inheritance and gene–environment interactions, particularly involving familial and social influences^16^.

Genome-wide association studies (GWASs) have emerged as powerful tools for identifying common genetic variants associated with traits and diseases^17,18^. Genetic associations with alcohol dependence have been mostly observed in genes involved in alcohol metabolism, including *ADH1B*, *ADH1C*, and *ALDH2*, as well as in genes affecting neurotransmission pathways, such as GABA receptor genes^19^.

A GWAS using data from 112,117 White British participants in the UK Biobank identified 14 loci associated with self-reported weekly alcohol consumption, including variants in the *ADH1B*, *ADH1C*, *ADH5* and *KLB* genes^20^. Genetic correlations were also observed between alcohol use and traits such as educational attainment, high-density lipoprotein cholesterol levels, smoking, and body size measures^20^. A more recent meta-analysis conducted by the GSCAN consortium leveraged data from up to 3.4 million individuals from four major ancestries, notably improving fine-mapping resolution and locus discovery, and identifying a novel association between number of drinks per week with *ECE2*^21^. The Million Veteran Program (MVP) has demonstrated that multi-trait GWAS approaches can increase discovery power, revealing novel pleiotropic variants associated with both alcohol use disorder (AUD) and smoking behavior^22^. Despite these advancements, most GWAS on alcohol consumption have been conducted predominantly in populations of European ancestry, with some notable exceptions. For example, a GWAS involving 3,132 African Americans (including 1,315 cases) identified significant associations on chromosome 3 related to alcohol dependence, including eQTLs for Nephrocystin 3 *(NPHP3)* in the sigmoid colon^23^.

In this study, we combined analyses from biobanks and population cohorts in Africa to expand investigations into the genetic determinants of alcohol use among underrepresented populations^24–27^. Here we explore and characterize genetic associations with alcohol drinking initiation and cessation among individuals of African ancestry, including both residents within the African continent and members of the African diaspora. By focusing on these two distinct yet complementary behavioral phenotypes, we aimed to identify genetic variants that may contribute to vulnerability or resilience in alcohol-related behaviors.

## Methods

### Studies and participants

The overall study design and analytical workflow are presented in Supplementary Figs. 1 and 2.

## AWI-Gen

The Africa Wits-INDEPTH Partnership for Genomic Studies (AWI-Gen), an H3Africa Collaborative Centre, is a population-based study with two waves of data collection^24,25,28^. AWI-Gen seeks to identify genetic and environmental factors that affect the changing disease burden and that contribute to cardio-metabolic disease in six African study sites in four countries^24^. The population-based study recruited ~ 12,000 participants from Ghana, Burkina Faso, Kenya and South Africa aged 40 to 60 years at baseline, collected blood and urine biomarkers, and captured extensive participant data, including patterns of alcohol consumption.

## JCS cancer

The Johannesburg Cancer Study (JCS) began in 1995 and concluded in 2016. Over the 20-year period, the JCS enrolled over 25,000 treatment-naive African cancer patients from the Greater Johannesburg region. Enrolled patients provided a blood sample and completed an in-depth lifestyle risk factors questionnaire, which included an assessment of drinking behavior^26^.

## UK Biobank

UK Biobank (UKB) is a longitudinal study in the United Kingdom including over 500,000 volunteers aged 40–69 years at baseline. Since 2006, it has collected extensive phenotypic and biological data to allow approved researchers to investigate the genetic and environmental determinants of a wide range of diseases and health-related phenotypes. We identified ~ 7,800 individuals of African ancestry in UKB using genotyped data and a clustering approach as described elsewhere^29^.

## Uganda Genome Resource

The Uganda Genome Resource (UGR) includes ~ 6,400 individuals and is a subset of the General Population Cohort (GPC), a population-based open cohort aimed at understanding HIV infections in Uganda. The UGR was built with the aim of improving local resources for public health and to allow large genetic epidemiology studies^30,31^.

### Genotyping and imputation quality control

The AWI-Gen and JCS participants were genotyped for ~ 2.2 M SNPs using the Illumina Infinium H3Africa SNP array, which is designed to capture common variants in African populations. Imputation resulted in ~ 17 M autosomal variants and was performed using the African Genome Resources reference panel (EAGLE2 + PBWT pipeline) through the Sanger Imputation Server. Details of quality control (QC) and imputation settings are presented elsewhere^32–34^.

Participants from UGR were in part genotyped for ~ 2.2 million autosomal markers using the Illumina HumanOmni2.5-8 BeadChip array (N = 4,778) and in part sequenced at low coverage (N = 1,978)^27,35^. Genotype imputation was conducted for ~ 98 million variants using a combined reference panel integrating whole-genome sequence data from the African Genome Variation Project^31^, the Uganda 2000 Genomes, and the 1000 Genomes Project Phase 3 (1000GP)^36^. Details on QC procedures and imputation settings are described elsewhere^35^.

Participants from UKB were genotyped at approximately 800,000 variants using the UK Biobank Axiom Array^37^. Genotype imputation was performed for ~ 93 million autosomal variants using a combined reference panel that included the Haplotype Reference Consortium (HRC)^38^, UK10^39^, and the 1000GP Phase 3^36^. Comprehensive details regarding QC procedures and imputation settings are available elsewhere^37^.

### Phenotype definitions

Two binary alcohol drinking traits were examined: Drinking Initiation (DI) and Drinking Cessation (DC). These traits were defined using relevant responses from questionnaire data available in each cohort. DI compares ‘never’ drinkers (controls) versus ‘ever’ drinkers (cases). ‘Never’ drinkers are individuals who have never consumed alcohol, while ‘ever’ drinkers currently drink alcohol or drank in the past. DC, on the other hand, includes only ‘ever’ drinkers and compares ‘current’ drinkers (cases), who were drinking every day or occasionally at the time of the questionnaire, to ‘previous’ drinkers (controls), who were not drinking at that time. Association testing for the DI phenotype was performed on AWI-Gen, UKB-AFR, UGR and JCS. DC could not be defined in JCS and in one of the sites in AWI-Gen South, and association analyses for DC included AWI-Gen, UKB and UGR. The number of cases and controls for each phenotype in each cohort is presented in Table 1 and Supplementary Fig. 2.

### Association testing

Genome-wide association analyses for JCS, UKB-AFR, and AWI-Gen were performed using a generalized mixed model, and significance was evaluated using SAIGE, which accounts for case-control imbalance^40^. Analyses for JCS and AWI-Gen were conducted using the H3A GWAS pipeline^41^. For UGR, associations were tested using a univariate linear mixed model (LMM), and significance was evaluated with a likelihood ratio test as implemented in GEMMA v0.98.1^42^.

Covariates included sex, age, age squared, and dataset-specific numbers of principal components (PCs): AWI-East, AWI-South and JCS (5 PCs), AWI-West (11 PCs), UGR (10 PCs), and UKB-AFR (9 PCs). PCs were computed separately for each dataset using principal component analysis of independent genotyped SNPs.

### Meta-Analysis

For each phenotype (DI and DC), a two-step meta-analysis was performed. In Step 1, we included all individuals from continental Africa, including AWI-East, AWI-South, AWI-West, JCS (stratified by cancer status), and UGR, resulting in two meta-analyses (DI_SSA_ and DC_SSA_). In Step 2, we meta-analyzed the results from Step 1 with data from non-continental African individuals, including UKB-AFR, resulting in two additional meta-analyses (DI_AA_ and DC_AA_). In each step, we used METAL to perform association analyses, following the H3AGWAS pipeline^41^.

Meta-analyses included variants found in at least two datasets, and genome-wide significance was considered at p < 5 x 10^− 8^ and suggestive significance at p < 5 x 10^− 6^. Sentinel variants were defined as those with the lowest p-value in a 1Mb region.

### LD reference panel

The linkage disequilibrium (LD) reference panel was constructed to capture the broad genetic diversity of the study population by combining data from 100 individuals from the AWI-Gen study (representing Southern African ancestry), the African Genome Variation Project (AGVP)^31^, and the AFR populations in the 1000GP^36^.

### Conditional analysis and Fine-mapping

Independence of sentinel variants was defined by using GCTA and performing a stepwise model selection of independently associated variants within ± 500 kb of each sentinel variant using the LD reference panel described above^43^. For each independent sentinel variant, we defined 99% credible sets with FINEMAP v1.4 using a shotgun stochastic algorithm assuming a single causal variant in a 1Mb window centered on the sentinel^44^.

### Variant annotation

Sentinel SNPs defined using the suggestive threshold, and their corresponding credible sets, were functionally annotated using ANNOVAR^45^. Gene-based annotations were performed with the refGene and ensGene databases to determine the functional context of each variant. Additional annotations were obtained by predicting functional impact from multiple algorithms (*dbnsfp42c*)^46^, clinical significance (clinvar_20221231), deleteriousness scores (cadd13)^47^, and allele frequencies from multiple population databases (popfreq_all_20150413), including 1000GP^36^, ESP6500^48^ and ExAC^49^.

### Phenome-wide association studies (PheWAS)

For the sentinel SNPs defined using the genome-wide and suggestive thresholds and their corresponding credible sets, we queried associations in the OpenGWAS database (https://gwas.mrcieu.ac.uk/, March 2025 release) using the *phewas* function from the ieugwasr R package^50^. Phenotypes were classified into categories using a semi-automated approach based on pattern matching. PheWAS plots were generated using ggplot2 (https://ggplot2-book.org/). A Bonferroni-corrected significance threshold of 5% was applied, adjusting for the number of sentinel SNPs identified at the suggestive threshold and the number of phenotypes retrieved from the OpenGWAS database (p < 0.05, N_sentinel_ SNPs × N_phenotypes_).

### Lookup in public datasets

Sentinel variants and their credible set defined at suggestive significance for all four meta-analyses (DI_SSA_, DI_AA_, DC_SSA_, and DC_AA_) were extracted from: (i) three datasets from the Million Veteran Program cohort, including alcohol use disorder (AUC [datasets pha004840.1 and pha004841.1]), Alcohol Use Disorder Identification Test-Consumption scores (AUDIT-C [datasets pha004844.1 and pha004845.1]) and Maximum Habitual Alcohol Intake (MaxAlc [datasets pha004848.1 and pha004849.1]) phenotypes in both African and European ancestries^51,52^; and (ii) one dataset from the GWAS & Sequencing Consortium of Alcohol and Nicotine use consortium, including Drinks per Week in both African and European ancestries^21^. Significance was evaluated by applying a Bonferroni correction on the number of variants tested for replication.

### Visualization results

Results were visualized using Manhattan and qq-plots using the fastman library^53^. Regional plots were created using LocusZoom standalone^54^.

### Mapping variants to genes and tofunctions

The Functional Mapping and Annotation of Genome-Wide Association Studies (FUMA) was used to annotate, prioritize, visualize, and interpret GWAS results^55^. GWAS summary statistics (p < 5 x 10^−5^) from our study were used as inputs. FUMA provided extensive functional annotation for all variants in genomic areas identified by lead variants in a biological context. We selected all candidate variants in the associated genomic region having r2 ≥ 0.6 (in 1000GP AFR populations) and MAF > 0.01 for annotation. Additionally, eQTL analyses were performed using publicly available datasets from several resources, including GTEx^56^ and PsychENCODE^57^.

Genes implicated by mapping of significant GWAS variants were further investigated using the GENE2FUNC option in FUMA, which provides hypergeometric tests of enrichment of the list of mapped genes in GTEx tissue-specific gene expression sets, MSigDB gene-sets, and chromatin states.

Multi-marker analysis of genomic annotation (MAGMA, v1.6) gene analysis was performed using LD in 1000GP AFR populations. Gene-based analysis enabled summarizing SNP associations at the gene level and association of the set of genes to biological pathways.

### Southern African Map of Blood Regulatory Variation (SABR)

We queried expression data from the South African Blood eQTL Resource (SABR) project. SABR is a map of blood regulatory variation derived from three South Eastern Bantu-speaking populations^58^. Using paired whole-genome sequencing and blood transcriptome data from over 600 individuals, we performed a two-step analysis. First, we screened all loci located within the 99% credible set of sentinel loci showing suggestive significance. Second, for loci reaching genome-wide significance, we analyzed the surrounding regions within a 150 kb window of each credible set; we used a p-adjusted FDR to determine significance at 0.05.

## Results

We present here the results of a genome-wide association study of two alcohol consumption phenotypes: drinking initiation (DI), comparing ever vs never drinkers, and drinking cessation (DC), comparing current vs past drinkers (Supplementary Fig. 2). The association analyses were conducted in two steps, first including only individuals living in continental Africa (DI_SSA,_ DC_SSA_), and then including individuals of African ancestry from the UK Biobank (DI_AA_, DC_AA_), for a total of four meta-analyses (Fig. 1 and Supplementary Fig. 3).

### Genome-wide significant loci

Two genomic variants reached genome-wide significance (Table 2, Supplementary Fig. 4). The first sentinel variant was observed on chromosome 1 (rs17114386, p = 4.49E-08), located in an intron of the *FYB2* gene, and was significant in the DC_SSA_ analysis. Previous GWAS have reported associations in the 1Mb region around this variant with behavioral traits such as smoking initiation, educational attainment, cognitive performance, medication use, and cardiovascular or kidney-related phenotypes, including coronary artery disease and kidney function (Supplementary Fig. 4a and Supplementary Table 1).

### Suggestive associations

A total of 165 sentinel variants showed suggestive associations (p < 5E-06) including 46 in the DC_SSA_ phenotype, 39 in DI_SSA_, 39 in DC_AA_, and 41 in DI_AA_ (Supplementary Table 2 and Supplementary Table 3), distributed across 121 distinct genomic loci. Among 1,013 credible set SNPs, 717 unique variants were defined as part of the 99% credible sets of the suggestive sentinel variants across the four meta-analyses.

Genes mapped to suggestive signals for drinking initiation included *CSMD1, GRIA2, PLCL2, PTPRD*, and *DCDC2*. For drinking cessation, mapped genes included *ADGRB1, AUTS2, CAMKK1, CPNE3, FAIM2, GJA1, KCNQ1, RORA, SLC6A17*, and *TSNARE*.

Among the SNPs within the credible sets, 28 variants had CADD PHRED scores greater than 13, including five sentinel SNPs (rs116196160 p = 1.2E-7, rs16892629 p = 2.8E-6, rs4943939 p = 4.7E-7, rs1152488325 4.9E-6, rs17646883 1.0E-6). These variants are predicted to have a potentially deleterious impact on protein function and may represent putative causal variants (see Supplementary Tables 2 and 3).

### Expression analysis

Among the variants in the credible sets of the suggestive lead SNPs, 63 were associated with at least one significant eQTL in the SABR dataset after FDR correction. These loci implicated a total of 63 genes, including 36 genes at 26 drinking initiation loci and 29 genes at 27 drinking cessation loci (Supplementary Tables 3 and 4).

Comparison with additional GTEx^56^ and PsychENCODE^57^ resources identified 81 significantly regulated genes in whole blood and 23 in PsychENCODE. Most genes identified through the SABR analysis were also identified in GTEx, while these additional resources identified 74 genes that were not detected in SABR.

Among the genes identified through eQTL analysis using FUMA we found *CRTC3*, *ALDH5A1 or ASIC1.*

### Lookup in MVP

After lookups in the MVP cohort GWAS for alcohol traits, we observed that approximately 45% of the suggestive sentinel variants and their corresponding credible sets in our study were present in the MVP dataset, encompassing 84 genomic regions with at least one overlapping variant between our dataset and MVP summary statistics. No regions reached genome-wide significance after Bonferroni correction (p < 0.00059). However, when applying a more lenient significance threshold (p < 0.01), we identified several suggestive associations. A genomic region at chr1:213060204, initially identified in the DC_SSA_, showed association with the Alcohol Use Disorders (AUD) phenotype in the MVP dataset. Another region at chr3:24625546, also from DC_SSA_, was associated with AUDIT-C (p-min = 0.00164). In addition, regions at chr17:78075647 (from DI_SSA_) and chr12:12217076 (from DI_AA_) were associated with the MaxAlc phenotype at the same significance threshold (Supplementary Table 5).

### PheWAS

Using the 121 genomic loci identified through the suggestive significant SNPs, 40 credible set regions contained variants associated with traits in previous GWAS (p < 9.x10^−7^), including studies related to drinking initiation (Supplementary Table 6). For instance, rs75775827 has been linked to educational attainment, while rs75775827 and rs16900662 have been associated with dietary habits. Variants rs75636393, rs779381, and rs7118119 were previously associated with traits like impedance measures (waist-hip ratio), age at menarche, and related anthropometric traits (Supplementary Table 6).

Several windows included loci that were also linked to hematological traits. For example, rs79787369 and rs12536178 was associated with eosinophil count, rs80020206 with reticulocyte count, and rs12497413 with hemoglobin levels. Notably, rs33995115 is a highly pleiotropic SNP associated with a broad range of traits, including anthropometric, cardiovascular, hematological, and metabolic phenotypes (Supplementary Table 6).

In the context of drinking cessation, rs13258495 was found to have a strong pleiotropic effect, being associated with behavioral traits such as neuroticism, worry, sleeplessness, and irritability, as well as anthropometric traits like impedance and biomarkers such as alkaline phosphatase and systolic blood pressure. Additionally, rs116708028, rs4930651, and rs77213242 were associated with hematological, anthropometric, and metabolic traits respectively. In total, 18 credible sets were previously associated with more than one phenotype category, including neurological, behavioural, haematological, metabolic, cardiovascular, and anthropometric phenotypes (Supplementary Fig. 5 and Supplementary Table 6).

### Mapping variants to genes and to functions

In a gene-based analysis of DI (using a MAGMA threshold p < 2.6x10^− 06^), we found a significant enrichment of olfactory and sensory pathways (Supplementary Fig. 6). Our analysis also revealed a substantial overlap of gene sets with gene sets related to dietary habits such as macronutrient intake, pork consumption, oily fish consumption, response to cognitive-behavioral therapy in anxiety disorder, cooked vegetable consumption, non-oily fish consumption and processed meat consumption. For DC, the ethanol oxidation pathway was significantly enriched (Supplementary Fig. 7). DC also overlapped with gene sets related to general factor of neuroticism.

## Discussion

This study investigates genetic factors associated with alcohol drinking initiation and drinking cessation, two behavioral phenotypes that remain underexplored, particularly in populations of African ancestry. Prior genome-wide association studies (GWAS) have focused largely on alcohol consumption and dependence using phenotypes such as alcohol use disorder (AUD), alcohol use disorder identification test-consumption (AUDIT-C), maximum habitual alcohol intake^51,52^ and drinks per week^21^. These measures capture patterns of use and addiction but do not specifically address the behavioral and genetic factors underlying the decision to begin or stop alcohol use. By focusing on initiation and cessation, our study complements prior work and enables the exploration of genetic variants linked to behavioral resilience and recovery, which may be associated with different aspects of alcohol use behavior.

We investigated genetic associations with alcohol drinking initiation and cessation in sub-Saharan African populations and individuals of African ancestry in the UK. Our analyses identified two genome-wide significant loci associated with drinking cessation in sub-Saharan Africa and African ancestry individuals, and suggestive associations overlapping with loci previously reported for alcohol dependence, and hematological, anthropometric, and metabolic traits in global studies.

Our findings provide insights into the genetic architecture of both alcohol drinking initiation and cessation, incorporating evidence from previous GWAS, phenome-wide association studies (PheWAS), and gene expression analyses. The two genome-wide significant loci for drinking cessation were located on chromosome 1, in an intronic region of the *FYB2* gene, and on chromosome 16 near the *FTO* gene. While *FYB2* is not a known contributor to alcohol consumption or dependence, it has been reported as a potential prognostic biomarker for hepatocellular carcinoma, a disease in which alcohol is a major risk factor^59^. This locus is also in proximity to *PLPP3*, a gene implicated in cardiovascular conditions such as atherosclerosis^60^. The *FTO* gene plays a critical role in obesity and metabolic disorders^61^ and, in addition to its metabolic functions, has been associated with binge and problematic drinking, where its effects are modulated by sex, smoking status, and *ADH1B* polymorphic variants, particularly in individuals of European ancestry^62^. Furthermore, *FTO* has been linked to neuroticism and shows indirect effects on subjective well-being and problematic alcohol use^63^.

Several loci showing suggestive associations with drinking initiation have been previously implicated in alcohol-related and neurodevelopmental traits. These include *CSMD1*, *PLCL2, and PTRD* that have been associated with alcohol disorder. The *NRG1* gene shows suggestive significance and has been previously identified as a susceptibility factor for substance dependence^64^. Similarly, *DCDC2*, a gene involved in neural development, has been linked to reading disorders and cognitive functions^65^. For drinking cessation, *AUTS2* had been found previously to be associated with alcohol dependence^66^, while *RORA* or *GPM6A* were previously associated with smoking^21^.

Using SNPs from our four analyses, 40 SNPs were identified with CADD > 13, indicating that they may influence protein structure or gene regulation (Supplementary Table 3 and Supplementary Table 4). Among the variants included in the credible sets, 40 had CADD PHRED scores > 13, suggesting potentially deleterious functional effects. In the SABR)^58^ analysis, 43 genomic windows contained variants associated with significant eQTLs, involving 61 genes (Supplementary Table 4). These eQTL associations provide additional functional evidence for the interpretation of the identified loci. Among the 43 suggestive regions identified with SABR, we identified notable genes including *GSTM4* and *GPM6A*, both linked to alcohol dependence^67^. Other genes include *SGO1-AS1*, associated with psychiatric disorders, and *DZIP1L*, involved in kidney-related traits^68^, and *CCDC171* which has previously been linked to smoking behavior^21^. Using FUMA, genes with a change of expression relevant to alcohol used included *ALDH5A1* a coactivator of CREB-dependent transcription that participates in molecular pathways underlying reward learning and long-term neuroadaptations induced by alcohol exposure. *ALDH5A1*, which encodes succinic semialdehyde dehydrogenase, is involved in GABA metabolism, a neurotransmitter system central to the acute and chronic effects of alcohol^69^. Furthermore, *ASIC1* encodes an acid-sensing ion channel implicated in anxiety, reward processing, and responses to addictive substances^70^.

PheWAS analysis showed that 40 loci overlapped with previously reported associations, of which 18 were in pleiotropic regions where associations with multiple phenotype categories had been reported previously. For example, rs13258495 in the *MSRA* gene has been associated with blood regulation, neurological and behavioral traits and cardiovascular disease. Similarly, previous GWAS have found that rs77213242 has been linked to both cardiovascular and metabolic traits. The integration of genetic associations, gene expression, and PheWAS results provide additional context for the loci in the credible sets.

It has been reported that craving in alcohol drinkers was often triggered by chemosensory cues, such as taste and smell, which are linked to brain network connectivity. Our study showed significant enrichment of sensory and taste pathways. Data from the Human Connectome Project suggests that specific patterns of brain connectivity associated with taste and olfactory perception may serve as predictors of alcohol consumption behaviors in young adults^71^.

Multiple lines of downstream analyses provided evidence that some of the identified genetic signals may have broader biological effects beyond alcohol-related behaviours. The PheWAS identified associations across neurological, behavioural, haematological, metabolic, cardiovascular, and anthropometric traits, with 18 credible sets showing associations across more than one phenotype category. In addition, eQTL analyses identified regulatory associations for variants within several credible-set regions across multiple genes and expression resources. Together, these findings suggest that some of the loci identified in our study may have pleiotropic effects across biological and behavioural traits, providing additional context for the interpretation of their associations with drinking initiation and cessation.

The limited availability of large, harmonized datasets from different African regions hinders robust replication of findings. The Million Veteran Program (MVP), despite its scale, may not offer an ideal replication sample due to key differences in ancestry composition (primarily African American individuals with West African and European admixture), phenotype definition, and imputation coverage. The lack of dense representation of African genomic diversity in some imputation panels could further reduce the overlap of informative variants.

Future research with larger sample sizes, more granular phenotyping and contextual cultural and behavioral data, including expanded representation across understudied African populations will provide additional insights. Collaborative efforts that leverage unified protocols and cross-regional designs will be critical to strengthen the replicability and generalizability of findings. The incorporation of multi-omics data, environmental risk factors, and longitudinal designs will also enhance the biological interpretation of associations and clarify the causal pathways underlying alcohol initiation and cessation.

In conclusion, this study highlights the importance of investigating alcohol-related behavioral phenotypes beyond consumption and addiction, especially in diverse global populations. Our findings provide a foundation for future discovery and underscore the need for inclusive, well-powered studies to elucidate the genetic architecture of alcohol use behaviors in African populations.

## Supplementary Material

This is a list of supplementary files associated with this preprint. Click to download.

- Supplementarydrinkingdraft160826.docx
- SupplementaryTabledrinking160826.xlsx

## Figures and Tables

**Figure 1:**
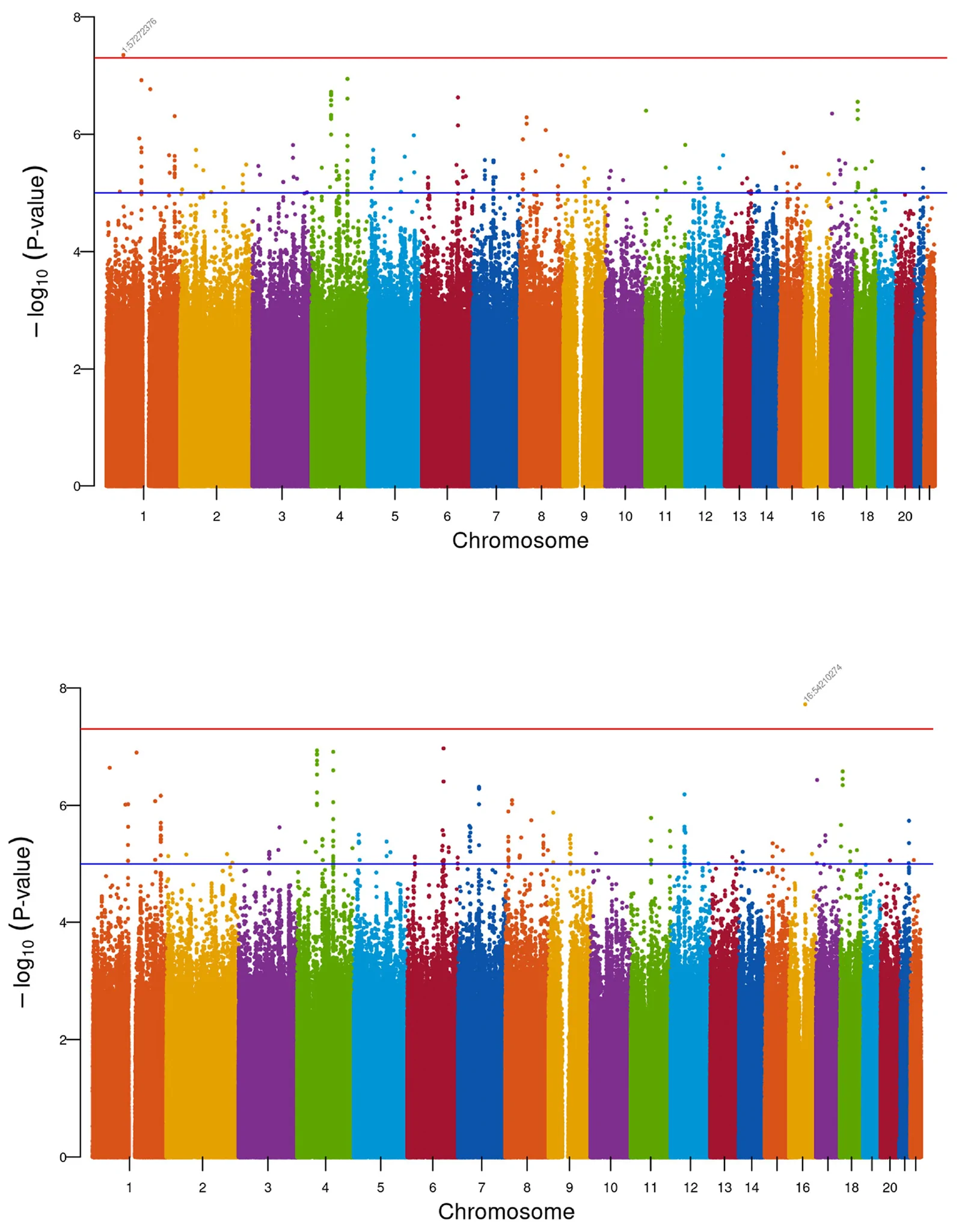
Manhattan plots showing the association results for DCAA (A), and DIAA (B). Lead SNPs reaching genome-wide significance (p < 5 × 10^−8^) are highlighted. The red line indicates the genome-wide significance threshold at p = 5 × 10^−8^, and the blue line marks the suggestive threshold at p = 5 × 10^−6^.

**Table 1:** Characteristics of participants included in the drinking initiation (DI) and drinking cessation(DC) genome-wide association analyses. Values represent sample size, percentage of females, and mean age (years) for cases (value 1) and controls (value 0) in each cohort. For DI, cases were defined as ever drinkers and controls as never drinkers. For DC, cases were defined as current drinkers and controls as previous drinkers.

Cohorts		Drinking Initiation		Drinking Cessation	
		Never (0)	Ever (1)	Current (1)	Previous (0)
**AWI-Gen**	**East**	899 (75.3%, 48.6)	819 (31.6%, 49.3)	309 (17.2%, 48.5)	510 (40.4%, 49.8)
	**South**	1,950 (71.1%, 54.6)	1,936 (25.6%, 54.5)	718 (27.9%, 56.5)	609 (48.6%, 57.3)
	**West**	747 (58.6%, 49.4)	2,786 (49.5%, 50.6)	2,267 (45.3%, 50.5)	519 (67.6%, 51.1)
**JCS**	**Soweto**	3,254 (94.8%, 45.9)	2,138 (76%, 48.5)	NA	NA
**UGR**	**Uganda**	4,261 (59.61%, 29.17)	2,146 (52.19%, 43.9)	1,659 (49.37%, 43.25)	487 (61.81%, 46.16)
**UKB**	**African ancestry individuals**	1,386 (68.04%, 51.56)	6,340 (55.88%, 51.91)	5,899 (56.43%, 51.87)	441 (48.53%, 52.43)
**Total**		**12,497 (72.6%, 42.6)**	**16,165 (52.1%, 50.3)**	**10,852 (50.0%, 50.5)**	**2,566 (53.3%, 51.6)**

Abbreviations: AWI-Gen, Africa Wits-INDEPTH Partnership for Genomic Studies; JCS, Johannesburg Cancer Study; UGR, Uganda Genome Resource; UKB, UK Biobank.

**Table 2:** Genome-wide significant associations with drinking cessation. Association results for the genome-wide significant loci identified in the meta-analyses of drinking cessation in sub-Saharan African populations (DCSSA) and individuals of African ancestry from the UK Biobank (DCAA). The table reports the chromosome (CHR), genomic position (BP, GRCh38), annotated gene or genomic context, lead variant (SNP), effect and other alleles, effect allele frequency, effect estimate, association P-value, and expression quantitative trait locus (eQTL) evidence from the Southern African Blood Regulatory Variation (SABR) resource. For SABR eQTL analysis, variants located within a ± 500 kb window around each genome-wide significant sentinel locus were evaluated. Genome-wide significance was defined as P < 5 × 10^−8^.

CHR	BP (hg19)	Genes	BP (hg38)	SNP	Effect allele	Other Allele	Effect	allele frequency	P-value	eQTL – Windows (SABR)
**DC_SSA_**										
1	57272376	*FYB2*, intronic	56806703	rs17114386	C	T	0.0752	−0.1289	4.49E-08	-
**DC_AA_**										
16	54210274	*FTO*	54176362	rs376814793	C	CTTAT	0.2517	0.081	1.90E-08	FTO

DCSSA, drinking cessation in sub-Saharan African populations; DCAA, drinking cessation in African ancestry populations; SABR, Southern African Blood Regulatory Variation.

The second variant was associated with DC_AA_ and it is located on chromosome 16 near the *FTO* gene ( rs376814793, p = 1E-08). Variants in the 1Mb region around this sentinel have been previously described as associated with alcohol-related traits, obesity, body fat and body size measurements, body mass index, and metabolic syndrome (Supplementary Fig. 4b and Supplementary Table 1).

## Data Availability

The GWAS summary statistics generated in this study will be deposited in the GWAS Catalog and will be made publicly available upon publication. Controlled-access individual-level genotype and phenotype data are available through the European Genome-Phenome Archive and other managed-access repositories, subject to approval by the relevant data access committees, as described below. JCS datasets are available through EGA under accession numbers EGAS00001007477 and EGAS00001008032. AWI-Gen genotype and phenotype datasets are available through EGA under accession numbers EGAD00010001996 and EGAD00001006425. Code and pipelines used for quality control and analysis are available at https://github.com/h3abionet/h3agwas/ and archived at Zenodo DOI 10.5281/zenodo.14907702. Additional analysis scripts are available from the corresponding authors.
